# Limited effectiveness of selected bioeffectors combined with recycling phosphorus fertilizers for maize cultivation under Swiss farming conditions

**DOI:** 10.3389/fpls.2023.1239393

**Published:** 2023-08-31

**Authors:** Sarah Symanczik, Carina Lipp, Paul Mäder, Cécile Thonar, Dominika Kundel

**Affiliations:** ^1^ Department of Soil Sciences, Research Institute of Organic Agriculture (FiBL), Frick, Switzerland; ^2^ Plant Genetics and Rhizosphere Processes Laboratory, TERRA Teaching and Research Center, University of Liège, Gembloux Agro-Bio Tech, Gembloux, Belgium; ^3^ Agroecology Lab, Université Libre de Bruxelles (ULB), Brussels, Belgium

**Keywords:** BIOFECTOR, bioeffectors, bacillus, field trial, humic acids, pseudomonas, seaweed

## Abstract

The use of plant biostimulants, also known as bioeffectors (BEs), has attracted increasing attention as an environmentally friendly strategy for more sustainable crop production. BEs are substances or microorganisms that are applied to plants or the surrounding soil to stimulate natural processes to enhance nutrient uptake, stress tolerance, and plant growth. Here, we tested the effectiveness of five BEs to enhance maize growth and phosphorus (P) uptake from various recycled P fertilizers in a series of pot and field experiments. First, the impact of two bacterial BEs and one soil-specific plant-based BE on crop performance was assessed in a 4-week screening experiment conducted in two arable, P-deficient soils of differing soil pH (a silty clay loam of pH 7.1 and a silty loam of pH 7.8) amended with recycled P-fertilizers (rock phosphate, biogas digestate, green waste compost, composted dairy manure, and chicken manure pellets). Then, for each soil type, the plant growth-promoting effect of the most promising BE–fertilizer combinations was re-assessed in an 8-week experiment. In addition, over a period of up to 3 years, three field experiments were conducted with maize in which up to two bacterial BEs were used either alone or in combination with a plant-based BE. Our experiments show that while BEs in combination with specific P-fertilizers can promote maize growth within the first weeks of growth under controlled conditions, the observed effects vanished in the long term, both in pots and under field conditions. In a tracing experiment, in which we tested the persistence of one bacterial BE over a period of 5 weeks, we observed a drastic decrease in colony-forming units already 2 weeks after inoculation. As previously shown in other studies, our data indicate that the plant growth-promoting effects of BEs found under controlled conditions are not directly transferable to field conditions. It is suggested that the drastic decline in inoculated bacterial strains in the tracing experiment is the reason for the decline in plant growth effect.

## Introduction

Current agricultural practices rely on high input rates of synthetic fertilizers, pesticides, irrigation, and short-crop rotations (Tilman et al., 2002). This approach has led to a multitude of environmental problems including groundwater pollution, eutrophication of aquatic systems caused by soil erosion, nutrient leaching, and runoff (Tilman et al., 2001; Tilman et al., 2002). Synthetic fertilizer production and use also contribute significantly to greenhouse gas emissions, thus exacerbating climate change (Vermeulen et al., 2012). Additionally, soil processes can decrease the availability of some plant nutrients, such as phosphorus (P), leading to fertilizer inefficiencies and the need for surplus fertilizer application (Smil, 2000). Commonly used P fertilizers in conventional agriculture are manufactured from non-renewable resources with limited global reserves that are in addition concentrated in only a few countries. Therefore, effective recycling and judicious use of these resources are crucial for long-term sustainability (Smil, 2000; Cordell et al., 2009).

There is a growing interest in addressing the negative consequences of high-input agricultural practices, and extensive research is underway to find alternative ways to produce food in a sustainable and eco-friendly manner. Various methods have been explored to minimize fertilizer inputs in agroecosystems, such as breeding plants with superior P-uptake efficiency (Lynch and Brown, 2001), using specific fertilizer placement techniques (Dunbabin et al., 2009), and utilizing soil microorganisms and natural extracts that possess properties that enhance plant growth and nutrient acquisition (Adesemoye and Kloepper, 2009).

In the last decades, the adoption of beneficial microbes or active natural metabolites, known as bioeffectors (BEs), has gained popularity as a sustainable way to increase crop productivity and improve plant health, thereby reducing the use of agrochemicals in crop production systems (Backer et al., 2018). BEs are substances or microorganisms that, when applied to plants or the surrounding soil, stimulate natural processes to enhance nutrient uptake, stress tolerance, and plant growth. BEs are different from fertilizers in the sense that they do not directly provide nutrients to plants, but instead, they enhance the plant’s ability to absorb and utilize nutrients. BEs can be derived from a variety of natural sources and can be categorized into two main types, microbial and non-microbial BEs (Du Jardin, 2015). Microbial BEs are beneficial microorganisms, such as bacteria and fungi that colonize the plants’ rhizosphere or endosphere and promote plant growth and health through various mechanisms: Microbial BEs can enhance plant growth directly by producing phytohormones (Backer et al., 2018) or indirectly by producing a variety of enzymes that solubilize P and potassium (K) in the soil thereby making nutrients more available for plant growth (Wozniak et al., 2020; Sible et al., 2021). Non-microbial BEs are mainly plant extracts gained from a variety of natural sources, including humic acid or extracts from seaweed. Humic acids can increase nutrient availability by chelating micronutrients in the soil and enhance plant growth by stimulating root development and promoting plant metabolism (Jindo et al., 2020; Yang et al., 2021; Herrmann et al., 2022). Moreover, humic acids can promote microbial activity in the soil, which can enhance nutrient cycling and improve soil health (Yang et al., 2021). Seaweed extracts further contain natural growth hormones, such as auxins, cytokinins, and gibberellins that may stimulate plant growth and development (Mukherjee and Patel, 2020). They also contain trace elements, including iron, zinc, and manganese, that are essential for plant growth and development.

BEs, especially microbial BEs, have been extensively studied for their efficacy on diverse crops in various ecosystems, resulting in numerous publications summarizing their benefits. However, when applied by farmers in practice, the expected effect often fails to materialize mainly due to environmental factors, soil conditions, fertilization practice, type of BE, and crop cultivar (Schütz et al., 2018). Fertilization, i.e., the type and amount of fertilizer applied, can impact the effectiveness of BEs. Some BEs may work better in conjunction with reduced amounts of synthetic fertilizers, or in systems that incorporate organic fertilizers (Thonar et al., 2017). Similarly, soil properties, such as pH, organic matter content, and nutrient availability, were shown to have a strong impact on the effectiveness of BEs as well as the interaction between BEs and the soil microbiome (Mosimann et al., 2017). Given the complexity of these factors, it is important to carefully consider the use of BEs in a specific agricultural system and to ensure that they are applied in a way that maximizes their effectiveness. Hence, further research is required to determine the specific conditions that enable BEs to enhance plant growth more consistently and predictably. This information can be used to develop tailored BEs that could increase fertilizer efficiency and reduce agriculture’s reliance on synthetic fertilizers.

To assess the effectiveness of five BEs to enhance maize growth and P-uptake from various recycled P fertilizers, we conducted a series of pot and field experiments. First, we performed a screening experiment with a combination of BEs and recycled P fertilizers in soils of differing pH. Then, for each soil type, the plant growth-promoting effect of the most promising BE–fertilizer combinations was re-assessed in a follow-up experiment. In addition, over a period of up to 3 years, three field experiments were conducted with maize in which up to two bacterial BEs were used either alone or in combination with non-microbial BEs, consisting of humic acids or algal extracts. To investigate the factors explaining the observed results, we assessed the persistence of one bacterial BE in a tracing experiment and the effects of humic acids on soil properties. This study was conducted as part of the European project BIOFECTOR (7th FP), which focused on reducing the use of mineral fertilizers in European agriculture. The project aimed to develop adapted BEs that can enhance the efficiency of alternative fertilization approaches, including organic farming, low-input farming, and the utilization of fertilizers derived from recycling waste products.

## Materials and methods

### Experimental design

Maize growth (variety Colisée, KWS Saat, Germany) was investigated in pots using topsoil collected from two fields of different pH and management: “Buus” soil was collected from an organically managed arable field low in soil P content and neutral pH (pH_H2O_ = 7.1 and “Dompierre” soil from an alkaline calcareous grass clover lay (pH_H2O_ = 7.8). In addition, field experiments were conducted on two organically managed farms: the “Buus” site (47°30′42.9″N 7°50′50.0″E, Basel-Land, Switzerland), from where also soil for experiments under controlled conditions was collected, and the “Hagenwil” site (47°31′35.4″N 9°18′28.1″E, Thurgau, Switzerland) where an on-farm experiment was conducted in collaboration with the farmer. For more details on soil properties, see **Table 1**. Experiments were established following a factorial design including up to three factors: P fertilization, microbial BE application (BE), and soil-specific, non-microbial BE application (from here on referred to as additive). Pot experiments were conducted with different organic recycled P fertilizers and with two mineral fertilizer controls. **Table 2** gives an overview of the experiments and the tested factors applied in each experiment.

**Table 1:** Properties of soils.

Soil/origin	Management	Soil type	Texture			Soil pH_H2O_	Organic carbon (%)	Phosphorus (P)
			Clay	Sand	Silt			Olson^a^ DL^b^ (mg P/kg)
			(%)	(%)	(%)			
Buus	Organic arable field	Silty clay loam	29.9	3.90	66.2	7.1	2.64	6.5^a^
Dompierre	Conventional grass clover lay	Silty loam	14.8	43.5	41.7	7.8	1.26	10.3^b^
Hagenwil	Organic arable field	Silty loam	19.7	29.3	51.0	6.5	2.45	1.82^a^

DL, Double lactate.

**Table 2:** Overview of the experimental design and setup of the experiments under controlled conditions (Exp. 1–4) and field conditions (Buus1,2; Hagenwil).

	Exp. 1	Exp. 2	Exp. 3	Exp. 4	Buus1	Buus2	Hagenwil
Factors tested:							
Bioeffectors							
No bioeffectors (noBE)	x	x	x		x	x	x
Proradix	x	x	x		x	x	x
Rhizovital					x		x
BEmix	x	x					
Additive							
No additive (A0)	x	x	x	x		x	
Nematec	x		x			x	
Humic acids		x		x			
Fertilizers							
No P fertilizer (noP)	x	x	x	x	x	x	
Triple superphosphate	x	x	x	x			
Rock phosphate	x	x					
Digestate	x	x		x			
Green waste compost	x	x		x	x		
Dairy farmyard manure	x	x					
Chicken manure pellets	x	x	x	x		x	
Experimental setup:							
Growing conditions	CC	CC	CC	CC	Field	Field	Field
Soil origin/location	Buus	Domp	Buus	Domp	Buus	Buus	Hagenwil
Substrate per pot (kg DW equivalent)	1	1	2.5	2.5	na	na	na
Growth period (weeks)	4	4	8	8	17, 18	17	24, 26, 27
Number of seasons	na	na	na	na	2	1	3
Number of replicates	4	4	5	5	4	4	4

CC, controlled conditions; Domp, Dompierre; na, not applicable; DW, dry weight. x indicates the selection of variants for a given experiment.

### BE and additive treatments

Three microbial BE products and two soil-specific additives were tested in total. These BE treatments included the following: Proradix WP (Sourcon Padena, Germany) containing *Pseudomonas* strain DSMZ 13134 (Proradix), RhizoVital 42 fl. (Abitep, Germany) containing *Bacillus amyloliquefaciens* strain FZB42 (RhizoVital), and BEmix containing *Bacillus licheniformis*, *B. megaterium*, *B. pumilis*, *B. subtilis*, *Paenibacillus polymyxa* with >10^9^ colony-forming units (CFU)/g product for each bacterial strain, *Trichoderma harzianum* strain OMG08 with > 10^10^/g product, and 15 mg of Mn/Zn per gram product. While *Trichoderma* belongs to the fungal kingdom, all other microbial BEs used are bacteria. The initial project experiments have shown that the majority of these components are effective in enhancing crop growth. Thus, the BEmix was newly formulated by partners of BIOFECTOR to be tested within the project. The choice of Proradix and RhizoVital is based on their published ability to promote maize growth under similar soil conditions and fertilization strategies (Thonar et al., 2017). For BE application, BE suspensions were prepared under sterile conditions by diluting the products with 2.5 mM CaSO_4_/water in pot/field experiments and inoculating at a concentration of 2 × 10^6^ CFU per gram of substrate/soil. Additive treatments included either AgriPrime Nematec® (BioAtlantis Ltd., Ireland) containing *Laminaria digitata* (Nematec), a derived-brown alga product, applied to the microbial-rich Buus soil or humic acids extracted from artichoke residue compost (Monda et al., 2018) applied to the alkaline Dompierre soil. Nematec was selected based on the producers’ experience that Nematec stimulates microbial grazers that can improve crop nutrient supply in microbe-rich soils such as the Buus soil. Humic acids were selected based on preliminary project results of improved P supply from recycled fertilizers in alkaline soils. Non-inoculated controls (noBE/A0) were included in each experiment testing BEs/additives. Further details on BE and additive application are given in **Supplementary Data 1.1**. All BEs and additives were provided by the EU-BIOFECTOR project partners and additional information on the BIOFECTOR project and the BEs used is available on the website: http://www.biofector.info.

### Fertilization treatments

Fertilization treatments included several organic recycled P fertilizers: biogas digestate (Leureko, Rheinfelden, Switzerland) with a P content of 0.21%, sieved at 10 mm and referred to as digestate; compost from green waste (Leureko, Rheinfelden, Switzerland) with a P content of 0.281%, sieved at 10 mm and referred to as compost; composted dairy farmyard manure with a P content of 0.64% and referred to as FYM; and pelleted chicken manure (Agriges, Italy) with a P content of 1.7%, ground and sieved at 1 mm and referred to as pellets. In addition, rock phosphate (Sebald Zement GmbH, Germany) with a P content of 11.1%, ground and sieved at 1 mm and referred to as RP, and Triple Superphosphate with a P content of 20%, ground and sieved at 1 mm and referred to as TSP, were partly included as positive controls. A non-P fertilized control (noP) was included in every experiment testing different P fertilizers. Except for noP treatments, pots received P at a dose of 50 mg of P/kg of dry substrate and field plots at a dose of 50 kg of P/ha.

### Experimental setup

#### Growth experiments under controlled conditions

The four experiments under controlled conditions followed a fully randomized design with four replicates (4-week screening experiments: Exp. 1 and Exp. 2) and five replicates (8-week growth experiments: Exp. 3 and Exp. 4) per treatment. After sieving, the soil was mixed with quartz sand (0.6–1.2 mm) in the ratio of 2:1 [soil dry weight (DW)/sand]. Each pot contained the equivalent of 1 kg or 2.5 kg DW of the experimental substrate (**Table 2**). Besides P fertilizers specified in **Table 2** and above, all pots received nitrogen (N) (100 mg of N/kg substrate) and potassium (K) (166 mg of K/kg substrate) in the form of calcium nitrate and potash magnesia, respectively. Where organic recycled P fertilizer containing N and K were applied (**Table 2**), the basal dose of mineral N and K fertilizers was reduced accordingly. The N, K, and P fertilizers were homogeneously mixed into the substrate before potting. Water addition was adjusted to reach 60–70% of the substrate’s maximal water-holding capacity (WHC). Three seeds were sown per pot, and the BE suspension or water (for noBE treatments) was added to the seeding hole. After covering the seeds with the substrate, additive suspension or water (for A0 controls) was applied on the surface at a distance of 5 cm surrounding the seed. The surface of the pots was then covered by a fine layer of quartz sand to avoid the formation of surface crusts after watering and pots were covered with plastic foil until germination of maize to avoid water loss due to evaporation. The pots were randomly placed into a growth chamber with 12-h day/12-h night, 26/22°C, 30,000 lux (mercury/natrium lamps), and 60% relative humidity and watered according to the plants’ needs to keep the initial water holding capacity (WHC) of 60% (increased to 70%–80% after 2 weeks). Thinning (including the root systems) was performed 1 week after sowing, leaving one plant in each pot. A second and third application of Nematec/humic acids and Nematec, respectively, were conducted (**Supplementary Data 1.1**). During the growth period, plant height and stem diameter were measured and the final harvest took place 4 or 8 weeks after sowing by cutting the plants shortly above the soil surface. Fresh weight was measured before plants were dried at 60°C to determine the shoot DW and milled for elemental analyses. The shoot P-concentration was measured using the molybdate blue method (Murphy and Riley, 1962) on a Segmented Flow Analyzer (Skalar Analytical B.V., San++ Automated Wet Chemistry Analyzer, Breda, Netherlands) after incineration and acid extraction of the shoot powder. The root system was washed from the substrate, weighed, and dried to determine the total root DW.

#### Field experiments at the Buus site

At the Buus site, two experiments were conducted (**Table 2**). The selection of BE treatments of the trial “Buus 1” was based on the results presented by Thonar et al. (2017), which showed in a pot experiment that the two BE products Proradix and RhizoVital enhanced maize growth combined with organic fertilizers. To further validate this potential, we tested these two Bes combined or not with compost at the Buus site for two consecutive years (2014 and 2015). The selection of treatments for the trial “Buus 2” was based on results observed in the screening experiment using the Buus soil (experiment 1). Both field experiments were designed in randomized blocks with four replicated plots (single plot size 3 m × 8 m) per treatment. N and K were applied at a rate of 110 kg of N/ha and 220 kg of K/ha in the form of potassium sulfate (33.2% K, Landor, Schweiz) and horn meal (15.4% N, Hauert, Schweiz). Where P fertilizers containing N and K were applied, the basal dose of N and K was reduced accordingly. The N, K, and P-fertilizer composts were homogeneously spread over the plot after plowing and incorporated with a rotary harrow. Maize seeds were sown manually into seeding furrows of 10 cm depth and inoculated with BE suspension or treated with the same volume of water (noBE treatments) before closing the seeding furrow **Supplementary Figure S1A**). For plots receiving the additive Nematec, the diluted product was applied above the seeding furrow. To mimic under-foot fertilization, chicken manure pellets were spread manually in 15- to 20-cm-deep strips between the maize rows. A second BE application was conducted at the two-leaf stage and a second and third application of Nematec were conducted at the two- and five-leaf stage, respectively (for more details, see **Supplementary Data 1.1**, **Supplementary Figure S1B**). Total shoot biomass was harvested at the reproductive stage (R3–4) cutting the shoots 5 cm above the ground before the fresh weight was determined. Subsamples of the biomass were then dried at 60°C to calculate shoot DW and further milled for elemental analyses (as described above).

#### Field experiment at the Hagenwil site

The three on-farm experiments, conducted in 2014, 2015, and 2017, were arranged in a randomized strip design with four replicated strips (min. 150 m) per treatment. In these experiments, only BEs previously reported to enhance maize growth in combination with organic fertilizers (Thonar et al., 2017) were tested and compared. Thus, fertilization was the same for all strips and consisted of sheep manure (20 t/ha) and chicken or pork slurry (14 t/ha) in 2014, and sheep manure (3 t/ha) in 2015 and 2017 and spread according to farmers’ practice before sowing. The BE suspensions or water (noBE treatments) were applied during sowing with a specially converted seeding machine and a second time at the three-leaf stage during mechanical weeding with a specially converted weeding machine (**Supplementary Figure S1C, D**; **Supplementary Data 1.2**). Using this innovative technique, BEs were applied directly to the maize seed in the drilling furrow. At full maturity (R6), plant density was determined from each of the two subplots and the corncobs harvested to assess the number of corncobs and their total FW. Ten representative corncobs were selected and further analyzed in the lab. The corn yield was calculated by removing the grains from the cobs of all subplots and drying both parts to assess their DW to calculate the total corncob yield.

### Tracing experiment

To assess the persistence of *Pseudomonas* DSMZ 13134 contained in the product Proradix, a tracing experiment was conducted. Pots were filled with potting substrate, and maize seeds were sown and inoculated with Proradix. At five time points, each of the three pots was harvested. The persistence of the bacterial inoculum was determined by qPCR. The setting up of the experiment was in principle identical to that described for Exp. 3 in *section 2.4.1*. Fifteen 3-L pots were filled with the Buus soil and fertilized with pellets. Per pot, three maize seeds were sown and inoculated with Proradix. All pots were randomly placed on a table and randomized twice and once a week during the first and following weeks, respectively. One week after sowing, the first three pots were harvested. In the following 4 weeks, each of the three pots was harvested every week. Harvest was performed by cutting the stems directly at the soil surface and the FW was determined. After drying stems at 105°C for 12 h, the DW was assessed. The root system was carefully freed from soil and washed in a water bath. Roots were carefully dried with paper towels and the root FW was recorded. A subsample of fine roots was taken and stored at −20°C for extraction of DNA. The remaining part of the root system was dried at 105°C for 12 h before the root DW was determined. DNA extraction and qPCR analyses to quantify *Pseudomonas* DSMZ 13134 in the rhizoplane were performed as described in Mosimann et al. (2017).

### Soil incubation experiment

This experiment was set up to exploit the potential of humic acids concerning its potential to promote P mobilization and the activity of microorganisms. The experiment included the same treatments as described for Exp. 4 with four replicates each. The preparation of the substrate was the same as described in *section 2.4.1*, but instead of 3-L pots, the substrate was filled in 0.5-L pots, placed into boxes, and incubated in the climate chamber for 8 weeks at 26/22°C (day/night). At the beginning and after the incubation period of 8 weeks, soil pH, resin-extractable P (resin P), and respiration of the microorganisms in the soil (soil basal respiration) were measured (for details concerning the methods, see **Supplementary Data 1.3**).

### Statistical analyses

All data were analyzed in R version 4.2.2 (R Core Team, 2022) through Rstudio version 2023.3.0.386 (RStudio Team, 2020), and graphs were produced using *ggplot2* (Wickham, 2016). Using linear models, the effects of the factors “bioeffector,” “additive,” and “fertilization,” as well as all of their two- and three-way interactions, were examined. Data from the field experiments (Buus 1, Buus 2, and Hagenwil) were analyzed using linear mixed-effect models through the function *nlme::lme* (Pinheiro et al, 2022 and R Core Team, 2022). We included the experimental factors as shown in **Table 2** as well as all possible interactions in the model. The random factors were chosen to model the spatial and/or temporal independency of the collected data and consisted of random = ~ 1|Year/Block (Buus 1), random = ~ 1|Block (Buus 2), and random = list (Year = ~1, Block = ~1 | Year, Rep_Strip = ~1 | Block, Rep_inside.strip = ~1 | Rep_Strip) (Hagenwil). The statistical significance of the main effects in the mixed models was derived using the *anova* function. Estimated marginal means for the factors explaining a significant amount of variation in the data were, for all models, derived through the function *emmeans::emmeans* (Lenth, 2023). If the factors “bioeffectors” or “additive” (or any of the interactions involving these factors) explained a significant amount of variation in the data, we conducted the corresponding *post-hoc* tests and generate pairwise mean comparisons using the *emmeans::emmeans* (Lenth, 2023) with Bonferroni-adjusted *p*-values. All models’ fit was visually verified, and if necessary, data were transformed to conform to the model residuals’ variance homogeneity and normal distribution assumptions. Data analysis is available as an Rmarkdown script (https://zenodo.org/record/8169013).

## Results

### Plant growth experiments under controlled conditions

We conducted a total number of four plant growth experiments to evaluate the efficacy of selected BEs to enhance maize growth under controlled conditions (see **Table 2** for details on the experimental setup).

**Figure 1:**
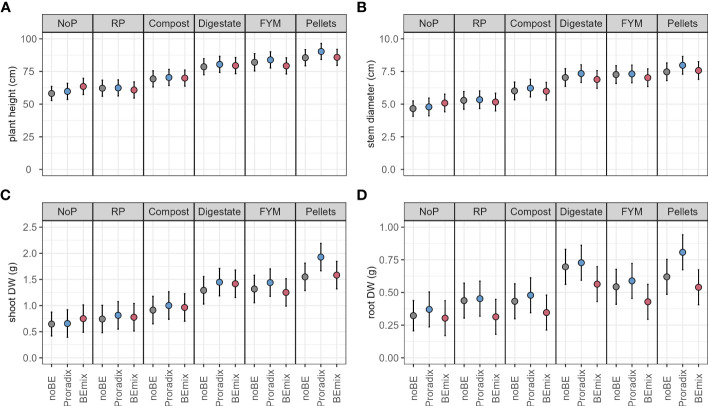
Model predictions (estimated marginal means) for experiment 1 with 95% confidence intervals of plant height **(A)**, stem diameter **(B)**, shoot **(C)**, and root **(D)** dry weight (DW) of maize inoculated with the bioeffectors (BE) Proradix or BEmix or without BE (noBE) and fertilized with rock phosphate (RP), compost, digestate, farmyard manure (FYM), pellets, or without P addition (NoP) assessed in experiment 1. Estimates are averaged over the levels of factor “Additive”.

In experiment 1, both BE application and fertilization explained a significant amount of variation in plant height, stem diameter, shoot DW, and root DW (**Figure 1**, **Table 3**, **Supplementary Table S1**). Conducting the corresponding *post-hoc* tests, we only found significant differences between levels of the factor BE application for the shoot and root DW. When compared to the control treatment without BE application, shoot and root DW increased with the application of Proradix (**Table 4**, **Supplementary Table S2**). Similarly, when compared to the control treatment noBE, root DW increased under the application of Proradix but decreased when the BEmix was applied (**Table 3**, **Supplementary Table S2**).

**Table 3:** Summary of significant treatment effects on plant growth parameters assessed in experiments 1–3 (Exp. 1–3) conducted under controlled conditions according to analysis of variance (ANOVA).

Experiment	Response	Source of variation	DF	Sum Sq	Mean Sq	*F* value	Pr(>*F*)
Exp. 1	Plant height (cm)	Bioeffector (BE)	2	260.137	130.069	3.979	0.021
	Stem diameter (cm)	BE	2	2.653	1.327	3.381	0.038
	Shoot dry weight (g)	BE	2	0.757	0.378	6.404	0.002
	Root dry weight (g)	BE	2	0.575	0.288	18.761	0.000
Exp. 2	Plant height (cm)	Additive	1	869.643	869.643	31.000	<0.001
	Stem diameter (cm)	Additive	1	7.809	7.809	26.278	<0.001
	Shoot dry weight (g)	Additive	1	0.352	0.352	8.947	0.003
		BE:Fertilization	10	3.920	0.392	9.960	<0.001
		Additive:Fertilization	5	1.525	0.305	7.748	<0.001
		BE:Additive:Fertilization	10	4.319	0.432	10.975	<0.001
	Root dry weight (g)	Additive	1	0.111	0.111	8.414	0.004
Exp. 3	Shoot dry weight (g)	BE:Fertilization	1	14.677	14.677	4.980	0.033

Degrees of freedom (DF), sum of squares (Sum Sq), mean squares (Mean Sq), p-value (Pr(>F)). For the complete table with all effects, see **Supplementary Tables S1**, **S3**, and **S5**.

**Table 4:** Mean comparisons of plant-related data with Bonferroni-adjusted *p*-values for experiments 1 and 2.

Experiment	Contrast	Estimate	SE	DF	*t*-ratio	*p*-value	Response
Exp. 1	Proradix–no Bioeffector (noBE)	1.908	1.163	111	1.640	0.311	Plant height (cm)
	BEmix–noBE	0.523	1.163	111	0.449	1.000	
	BEmix–Proradix	−1.385	1.167	111	−1.187	0.713	
	Proradix–noBE	0.206	0.127	112	1.630	0.318	Stem diameter (cm)
	BEmix–noBE	−0.002	0.127	112	−0.016	1.000	
	BEmix–Proradix	−0.208	0.128	112	−1.629	0.318	
	Proradix–noBE	0.138	0.049	112	2.800	**0.018**	Shoot dry weight (cm)
	BEmix–noBE	0.047	0.049	112	0.959	1.000	
	BEmix–Proradix	−0.090	0.050	112	−1.822	0.213	
	Proradix–noBE	0.062	0.025	112	2.483	**0.044**	Root dry weight (cm)
	BEmix–noBE	−0.093	0.025	112	−3.707	**0.001**	
	BEmix–Proradix	−0.155	0.025	112	−6.125	**<0.001**	
Exp. 2	Humic acids (HA)–no Additive (A0)	4.358	0.877	112	4.971	**<0.001**	Plant height (cm)
	HA–A0	0.394	0.090	112	4.372	**<0.001**	Stem diameter (cm)
	HA–A0	0.046	0.019	112	2.400	**0.018**	Root dry weight (g)

Standard error (SE), degrees of freedom (DF). Bold values highlight significant contrasts.

**Figure 2:**
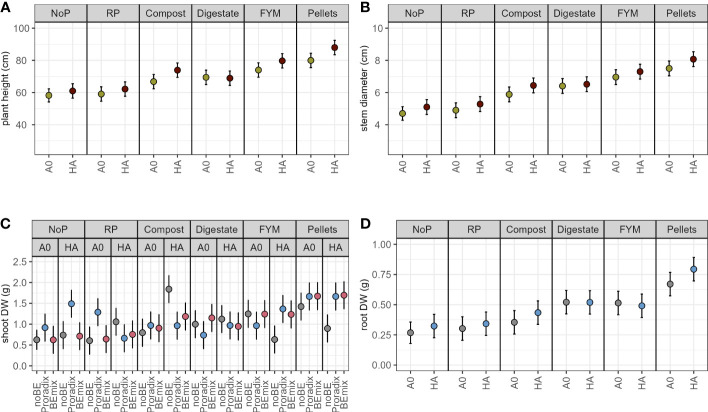
Model predictions (estimated marginal means) for experiment 2 with 95% confidence intervals of plant height **(A)**, stem diameter **(B)**, shoot **(C)**, and root **(D)** dry weight (DW) of maize inoculated with the bioeffectors (BE) Proradix or BEmix or without BE (noBE), supplemented with the additive humic acids (HA) or without additive (A0) and fertilized with rock phosphate (RP), compost, digestate, farmyard manure (FYM), pellets, or without P addition (NoP) assessed in experiment 2. Estimates in A, B, and D are averaged over the levels of factor BE.

In experiment 2, both the application of the additive and fertilization explained a significant amount of variation in plant height, stem diameter, and root DW (**Figure 2**, **Table 3**, **Supplementary Table S3**) with increased values when comparing the humic acid-treated plants to the control plants A0 (**Table 5**). For shoot DW, there was a complex three-way interaction between BE, additive, and fertilization (**Table 5**, **Supplementary Table S4**): When humic acids were combined with compost, BE application reduced shoot DW while BE application enhanced shoot DW when humic acids were combined with FYM or pellets. Under fertilization with digestate and when no humic acids were applied, shoot DW was lower for plants inoculated with Proradix compared to plants inoculated with the BEmix, while the opposite was found under fertilization with RP. The application of Proradix promoted shoot DW under no fertilization, both in the presence and in the absence of humic acids. When combined with RP and the application of humic acids, Proradix decreased shoot DW compared to the control without BE but it increased shoot DW when no humic acids were applied.

**Table 5:** Mean comparisons of maize shoot dry weight with Bonferroni-adjusted *p*-values for the screening experiment in experiment 2 conducted in the Dompierre soil under controlled conditions.

Contrast	Additive	Fertilization	Estimate	SE	DF	*t*-ratio	*p*-value
Proradix–no Bioeffector (noBE)	No additive (A0)	No phosphorus (noP)	0.291	0.121	112	2.399	0.054
Proradix–noBE	Humic acids (HA)	NoP	0.753	0.140	112	5.364	<0.001
BEmix–Proradix	HA	NoP	−0.778	0.140	112	−5.542	<0.001
Proradix–noBE	A0	Rock phosphate (RP)	0.682	0.140	112	4.865	<0.001
BEmix–Proradix	A0	RP	−0.645	0.140	112	−4.598	<0.001
Proradix–noBE	HA	RP	−0.395	0.140	112	−2.816	0.017
Proradix–noBE	HA	Compost	−0.875	0.140	112	−6.237	<0.001
BEmix–noBE	HA	Compost	−0.655	0.140	112	−4.669	<0.001
BEmix–Proradix	A0	Digestate	0.412	0.140	112	2.941	0.012
Proradix–noBE	HA	Farmyard manure (FYM)	0.732	0.140	112	5.222	<0.001
BEmix–noBE	HA	FYM	0.602	0.140	112	4.295	<0.001
Proradix–noBE	HA	Pellets	0.768	0.140	112	5.471	<0.001
BEmix–noBE	HA	Pellets	0.797	0.140	112	5.685	<0.001

Standard error (SE), degrees of freedom (DF). For the complete table with all mean comparisons see **Supplementary Table S4**.

In experiment 3, fertilization significantly affected plant height, shoot P uptake (mg P/pot), and root and shoot DW, while for the latter, there was also a significant interaction between BE application and fertilization (**Table 3**, **Supplementary Table S5**): When chicken manure pellets were used as fertilizer, there were no significant differences in shoot DW between the treatment group receiving Proradix and the control group without BE application (mean difference: 1.06, SE = 0.768, *p* = 0.176), while applying Proradix in the absence of P-fertilization marginally reduced plant DW when compared to the control (mean difference: −1.36, *p* = 0.085).

**Figure 3:**
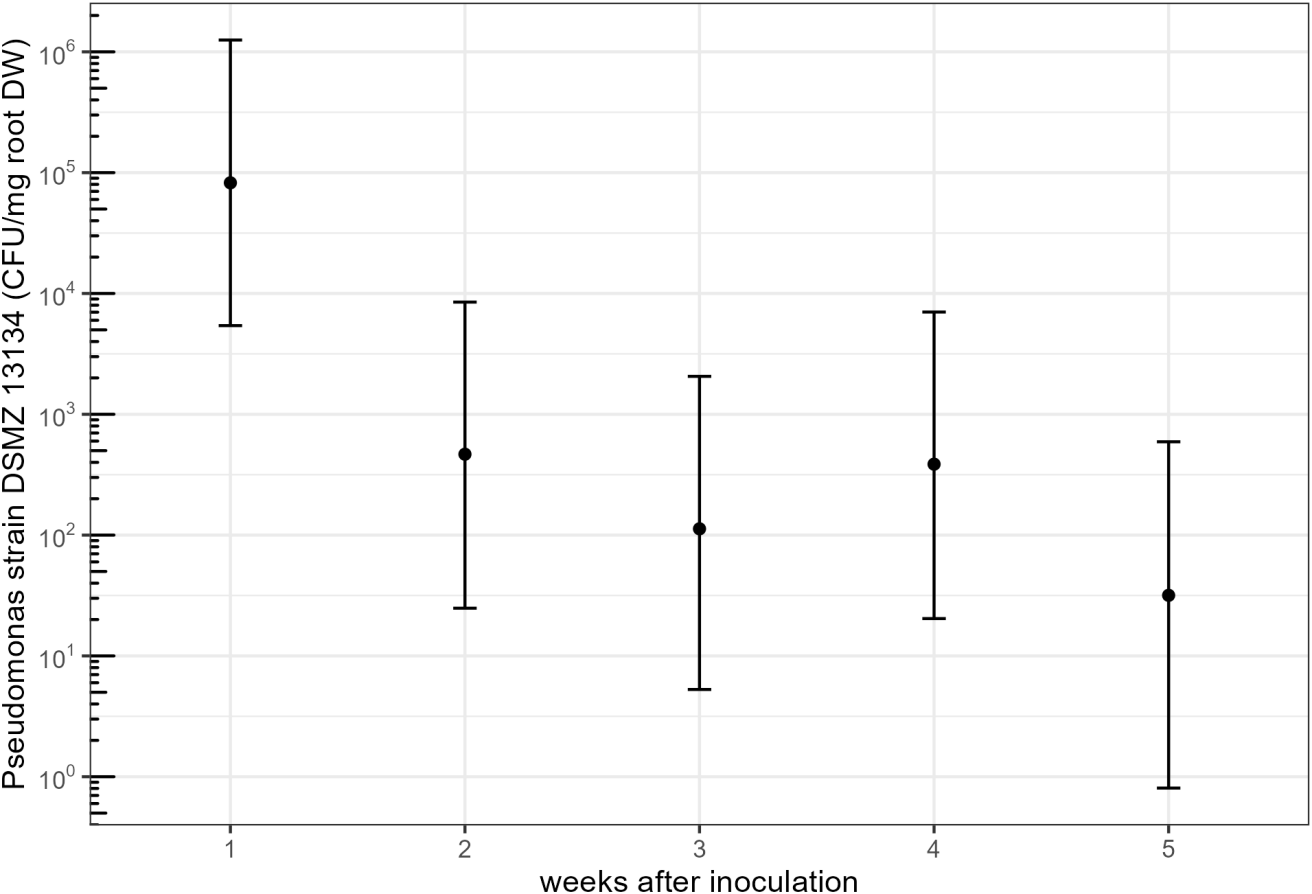
Model predictions (estimated marginal means) for the tracing experiment with a 95% confidence interval for colony-forming units (CFU) of *Pseudomonas* strain DSMZ 13134 detected per milligram of root dry weight (DW) over the first 5 weeks after inoculation. Data are log-transformed for graphical representation.

Using a qPCR-based tracing tool for *Pseudomonas* strain DSMZ 13134, the active ingredient of Proradix, we found the bacteria to be able to colonize the rhizosphere of maize roots; however, the abundance of *Pseudomonas* strain DSMZ 13134 changed significantly over time (F = 10.675, *p* = 0.001). One week after inoculation, the model-based prediction was 82,385 CFU/mg root DW, yet the number of CFU/mg root DW dropped significantly within the next week to 467 CFU/mg root DW (ratio = 0.006, SE = 0.01, *p* = 0.01), corresponding to a reduction in CFU/mg root DW of around 99% (**Figure 3**) and leveled off within the following weeks to values close to the detection limit (**Supplementary Table S6**).

In experiment 4, only fertilization but not additive application explained a significant amount of variation in the response variables (**Supplementary Table S7**). In the accompanying incubation experiment, there were no effects of humic acids on net soil pH, while for basal respiration and net resin P measured after 8 weeks, there was significant fertilization × additive interaction (**Supplementary Table S8**): Applying humic acids together with RP increased basal respiration when compared to the control without humic acids (ratio = 1.112, SE = 0.03, *p* = 0.001), but humic acids did not affect basal respiration when combined with any of the other fertilizers. The application of humic acids together with compost reduced the net resin *p*-value compared to the control (mean difference: −0.817, SE = 0.22, *p* = 0.001), while for the other fertilizers, it did not influence the net resin *p*-values whether humic acids were applied or not.

### Field experiments

In experiment Buus 1, neither the factor BE nor fertilization had a significant influence on the response variables investigated (**Supplementary Table S9**). In experiment Buus 2, there was a significant fertilizer × BE/additive interaction on plant height (**Supplementary Tables S10**, **S11**): Under fertilization with pellets and when compared to Proradix, the application of Nematec slightly promoted plant height (mean difference: 0.193, SE = 0.069, *p* = 0.043) while no difference between the two BEs was detected in the absence of fertilization (mean difference: 0.110, SE = 0.069, *p* = 0.401). In the experiment at Hagenwil, no effects of BE application were observed (**Supplementary Table S12**).

## Discussion

### Minimal and non-reproducible plant growth-promoting effects upon BE application under controlled conditions

We observed small and soil-specific plant growth-promoting effects when maize was grown with Proradix and humic acids in the Buus and Dompierre soil, respectively. However, even these small effects vanished when repeating the experiment in larger pots and extending the growth period from 4 to 8 weeks. Our results are in contrast with other previously published studies describing improved growth of maize and other crops after the application of BE products containing *Pseudomonas* strains or microbial consortia (Schütz et al., 2018; Bradáčová et al., 2020; Li et al., 2022). In particular, microbial consortia were often shown to have larger effects on crop growth than single strains (Kumar et al., 2016; Rubin et al., 2017; Herrmann et al., 2022). A reason for this might be that diverse consortia promote the survival and function of inoculated microorganisms and consequently establish more successfully in the soil compared to single-strain BEs as the likelihood of at least one strain escaping competitive exclusion is higher (Rivett et al., 2018). Moreover, the most pronounced effects of BEs were found in the dry tropics and the Mediterranean zone, with soils low in soil organic carbon (SOC) (Schütz et al., 2018). However, in our experiments, the growth promotion of maize could not be reliably observed with none of the tested BEs.

To reveal potential factors explaining the absence of a plant growth-promoting effect, we conducted a tracing experiment in the Buus soil, characterized by a high SOC. We observed that the *Pseudomonas* strain DSMZ 13134 was initially able to colonize the roots of maize, but was no longer detectable just 2 weeks after inoculation. Potential reasons for the inefficient persistence of the *Pseudomonas* strain DSMZ 13134 after initial establishment might be competition with the resident microorganisms, e.g., because of niche overlap, priority effects, facilitation (Hawkes and Connor, 2017), resource competition (Yang et al., 2017; Mallon et al., 2018), or predation through bacteriophages and microbial predators (Otto et al., 2017; Koskella and Taylor, 2018). Processes such as competition and predation are predominantly important in SOC-rich soils since SOC can support an abundant, diverse, and active microbial community (Lori et al., 2017). Also, Schütz et al. (2018) explained the low efficacy of P solubilizing BEs by high microbial activity resulting from elevated SOC, eventually hampering the establishment of the introduced BEs. This, in turn, could potentially diminish the effectiveness of the introduced BEs, similar to what we observed in our experiments with the SOC-rich Buus soil.

Besides SOC, various other abiotic factors were shown to influence the establishment and persistence of microbial BEs and, consequently, their efficacy to enhance crop growth. These include abiotic factors such as soil pH, soil texture, moisture, and salinity (Mäder et al., 2011; Mosimann et al., 2017; Schütz et al., 2018; Herrmann et al., 2022). Also, nutrient supply via fertilization was shown to determine the efficacy of BEs in promoting crop growth (Thonar et al., 2017; Mpanga et al., 2019; Weinmann and Neumann, 2020).

To determine the factors that explain a possible mode of action of the non-microbial humic acids in the alkaline Dompierre soil, we conducted an incubation experiment and tested whether humic acid application alters P availability (measured as resin P), soil pH, or microbial activity. We only found marginal and fertilizer-specific changes in microbial respiration and P-availability; effects that did not translate into a growth promotion of maize (data not shown). Our results are different from Yang et al. (2021) who collated the literature to explain potential modes of action by which humic acids can change various soil parameters including soil texture, cation exchange capacity, and water retention. The fact that we observed none or only minor fertilizer-specific changes upon humic acid application could point to an incompatibility between the HA and the selected organic fertilizers (Rose et al., 2014). Other possible explanations for the lack of observed effects in our plant growth and soil incubation experiments could be an inappropriate concentration of the HA solution applied or the timing of application (Rose et al., 2014). In addition, Dobbss et al. (2010) observed that differences in the sensitivity of plant species to humic acids influenced the success of humic acid application. Maize required twice the concentration of humic acids to stimulate root branching compared to the dicotyledon tomato and Arabidopsis, suggesting greater efficacy in monocotyledons.

### Lack of growth response under field conditions

Besides the limited efficacy under controlled conditions, we also did not observe any growth-promoting effects of maize upon BE application under field conditions. Owen et al. (2015) also reported poor reproducibility of commercial BEs under field conditions, and this, despite decades of research on the use and application of BEs. Similarly, Richardson and Simpson (2011) observed that the agronomic potential of BEs to promote maize yield was higher in pot experiments than under field conditions. Efficient root colonization is a prerequisite for many microbial BEs (Dobbelaere et al., 2001). As seen in our screening experiment, even at optimal and controlled conditions, the *Pseudomonas* strain DSMZ 13134 only transiently colonize the maize rhizoplane. Given this, it is unlikely to expect successful colonization under field conditions where additional stress factors with potentially negative impacts on the vitality of inoculants, root growth, and activity occur (Berg et al., 2021). As mentioned above, the high SOC contents of both field soils and the associated high microbial abundance and activity (Lori et al., 2017) may also have hindered the BEs’ potential to promote plant growth. We assume that this is the main reason why no stable plant growth-promoting effect was achieved in our experiments.

### Perspective of BE application

According to Schütz et al. (2018), BE application tends to be more effective in dry climates due to overall lower soil fertility, including lower levels of SOC, N, and P, resulting in lower abundance and activity of native soil microbes under these conditions. Furthermore, crops in dry climates are more likely to experience stress by factors like heat, drought, and salinity. By producing various molecules such as plant hormones, enzymes, and secondary compounds, microorganisms may help alleviate stress in plants ultimately leading to stabilized yields (Ali et al., 2022; Ma et al., 2022). Also, Rubin et al. (2017) found that microbial Bes are especially effective in promoting plant growth under drought. This is supported by a recent meta-analysis of Zhao et al. (2023) who observed increased plant biomass, enhanced photosynthesis, and inhibited oxidant damage under drought. Considering that in the future global dryland areas are expected to increase, BEs might become increasingly important.

## Conclusion

Although we did not observe any positive effects of BE application on soils and plants in the present study, we do not generally rule out the potential for BEs to positively affect plant growth and agricultural yields. However, our results highlight that factors including biological and chemical soil properties and climatic conditions play a fundamentally important role in determining the success of a BE application. In light of our results, we recommend against using BEs without conducting pretests. This applies particularly to arable cropping in temperate climates and fertile, SOC-rich soils often found in organically managed fields. It is crucial to carry out pretests specific to the crop, soil, and environmental conditions to identify effective products and mitigate the risk of financial losses.

## Data availability statement

The original contributions presented in the study are included in the article/**Supplementary Material**. Further inquiries can be directed to the corresponding authors.

## Author contributions

CT, DK, PM, and SS designed the experiment; PM, CL, DK, CT, and SS planned and conducted the experiment and performed the analyses; DK performed statistical analyses; DK and SS wrote the draft manuscript; and all authors revised the final manuscript. All authors contributed to the article and approved the submitted version.
