# Supplementary material for: Limited effectiveness of selected bioeffectors combined with recycling phosphorus fertilizers for maize cultivation under Swiss farming conditions
Source: Front Plant Sci. 2023 Aug 31;14:1239393. doi: 10.3389/fpls.2023.1239393 (PMC10501308; doi:10.3389/fpls.2023.1239393)
Supplement: Supplementary file 2 [file DataSheet_1.pdf]

Supplementary Material

Limited effectiveness of selected bioeffectors combined with recycling phosphorus fertilizers for maize cultivation under Swiss farming conditions

Sarah Symanczik[1*], Carina Lipp[1], Paul Mäder[1], Cecile Thonar[2,3], Dominika Kundel[1]

*** Correspondence:** Sarah Symanczik: sarah.symanczik@fibl.org

1 Supplementary Data

1.1 Application of bioeffectors and additives under controlled conditions and at field site Buus

Application of bioeffectors (BEs): BE suspensions were prepared under sterile/non sterile (experiments under controlled conditions/field experiment) conditions by diluting the products with 2.5 mM $CaSO_4$/water (experiments under controlled conditions/field experiment) and inoculated at a concentration of 2×10^6 colony forming units (CFU) per g of substrate/soil. In pots, BE suspension was applied into the seeding hole directly on top of the maize seed (5 ml per seeding hole = 15 ml per pot) and in field, BEs applied by band application into the seeding furrows (1st application at sowing) and to the stem base along the maize rows (2nd application two weeks after sowing).

Application of the additive Nematec (containing *Laminaria digitata* (Nematec), a derived-brown alga product, added to pots with Buus soil): On the sowing day, Nematec was diluted 444x in purified water (1 ml product + 443 ml purified water) and each pot received 12.5 ml of the diluted product or purified water (for controls without additive, A0). A sterile glass pipette was used to spread the diluted product or purified water at a distance of approximately 5 cm on the surface of the potting substrate. At leaf emergence, Nematec was diluted 222x (2ml product + 442ml purified water). Using a spraying bottle, the young leaves of each plant were sprayed with 12.5 ml of the diluted product or purified water. In the same way a third application was made seven days after leaf emergence using a 200x dilution (2.25 ml product + 222.75 ml purified water).

Application of the additive humic acids (HA, added to pots with Dompierre soil): The HA suspension was prepared by dissolving 0.6 g of HA in 1 ml 0.1M KOH. Then 1.2 L distilled water was added and the pH adjusted to 6.7. Then, the volume was topped up to 1.5 L with distilled water. On the sowing day, 5 ml of HA suspension or purified water (for controls without additive, A0) was applied into the seeding hole directly on top of the maize seed (5 ml per seeding hole = 15 ml per pot). Ten days after sowing, 15 ml of HA suspension or purified water (for controls without additive, A0) was distributed on the top of the surface using a pipette.

1.2 Application of bioeffectors at field site Hagenwil

First inoculation of BEs was conducted with a specially converted seeding machine: The drive and carrier machine is a John Deere 6620 tractor with 101.5 kW power. The soil cultivation is done by a blade rotor with 3 m working width. The tools between the rows are shortened and extended in the rows. This protects the soil because only as much soil is moved as necessary. In front of the extended tools, where the seeds subsequently come to rest, there is a cultivator coulter which runs slightly lower than the tools of the rotor. A reconsolidation roller runs behind the soil loosening where the maize rows come to rest. The sowing unit is a precision air seeder of the Kleine brand. In front of the sowing coulter runs a double disc coulter which opens the sowing slot. The spray nozzle is mounted in the sowing coulter behind the maize placement, which requires 1 litre of water at two bar per minute. The spray nozzle is fed from a tank at the front of the tractor. The pump for generating pressure is hydraulically driven via the oil connection at the front of the tractor. The whole device is mounted on a tooth packer roller.

1.3 Methods applied in the soil incubation experiment

1.3.1 Basal respiration

Immediately after mixing the additive and fertilizer with the substrate, 20 g DW equivalents of each mixture were weighted into perforated centrifuge tubes. Tubes were placed into 250 ml Schott bottles, filled with 41 g glass beads and 20 ml 0.05 M NaOH to cpature the released CO_2. Bottles were instantly closed with hermetic lids and transferred into an incubator at 25°C. After 24 h, the centrifuge tubes were transferred into a new set of Schott bottles, containing glass beads and 0.05 M NaOH. The bottles were closed and placed back in the incubator at 25°C. The previously incubated NaOH was titrated with a High-end-Titrator (808 Titrando, Metrohm AG, Herisau, Switzerland) under automatic addition of 1 ml 0.05 M $BaCl_2$ and 0.05 M HCl. The following six titrations over an incubation period of eight weeks were conducted after 2, 3, 10, 23, 37 and 57 days of incubation. Per titration, ten blanks were measured. The HCl consumption of each sample was recorded with the software "Tiamo" and the released CO_2 from soil microbes were calculated in µg CO_2-C/g soil/d, according to Equation 1. Data of the seven measurements were cumulated to assess soil respiration in µg CO_2-C/g soil.

$$BR = (B - S) \times \frac{2 \times (0.05 \times 6 \times 1000)}{t \times SW} \qquad (Equation\ 1)$$

BR	Basal respiration [µg CO_2-C/g soil/d]
B	Mean HCl consumption of the blanks [ml]
S	HCl consumption of the sample [ml]
2	Factor to convert 10 ml titrated NaOH in 20 ml employed NaOH
0.05	Concentration of HCl [mol/L]
6	Factor (1 ml 1 M HCl corresponds to 12 g/mol/2 CO_2-C) [g/mol]
1000	Factor to convert mg to µg
t	Incubation period [d]
SW	Soil weight [g soil – dw]

1.3.2 Soil pH_{H20}

Soil pH was measured in a 1:2.5 aqueous suspension at the beginning and after six weeks after incubation. In brief, 20 g DW equivalents of each additive-fertilizer-substrate mixture were weighted into 100 ml bottles. After adding 50 ml of demineralised water, the suspension was shaken manually and left for 12 h at room temperature (20°C). Immediately before the measurement, samples were well agitated and put on a magnetic stirrer. The pH was measured directly in the stirred suspension, by using a pH meter (inoLab® pH Level 1, WTW, Weilheim, Germany) with a glass electrode (SenTix® 81, WTW).

1.3.3 Resin-extractable phosphorus

The amount of available phosphorus (P) was estimated directly after mixing the additive and fertilizer into the substrate and after six weeks of incubation following a modified method of Nanzer et al. 2019. In brief, 8 g DW equivalents of each substrate mixture were weighted into 50ml centrifugation tubes. Then, 30 ml demineralised water and two anion-exchange resin membrane strips (AEM) were added and tubes were shaken for 24 h on a rotary shaker. Then, AEM were removed, rinsed with demineralised water and transferred into new 50 ml centrifugation tubes filled with 30 ml 0.1 M NaCl/HCl and shaken for 2 h to elute P absorbed on AEM strips. The P concentration of the extracts was measured using the molybdate blue method (Murphy and Riley, 1958) on a Segmented Flow Analyzer (Skalar Analytical B.V., San++ Automated Wet Chemistry Analyzer, Breda, Netherlands)

2 Supplementary Figures and Tables

2.1 Supplementary Figures

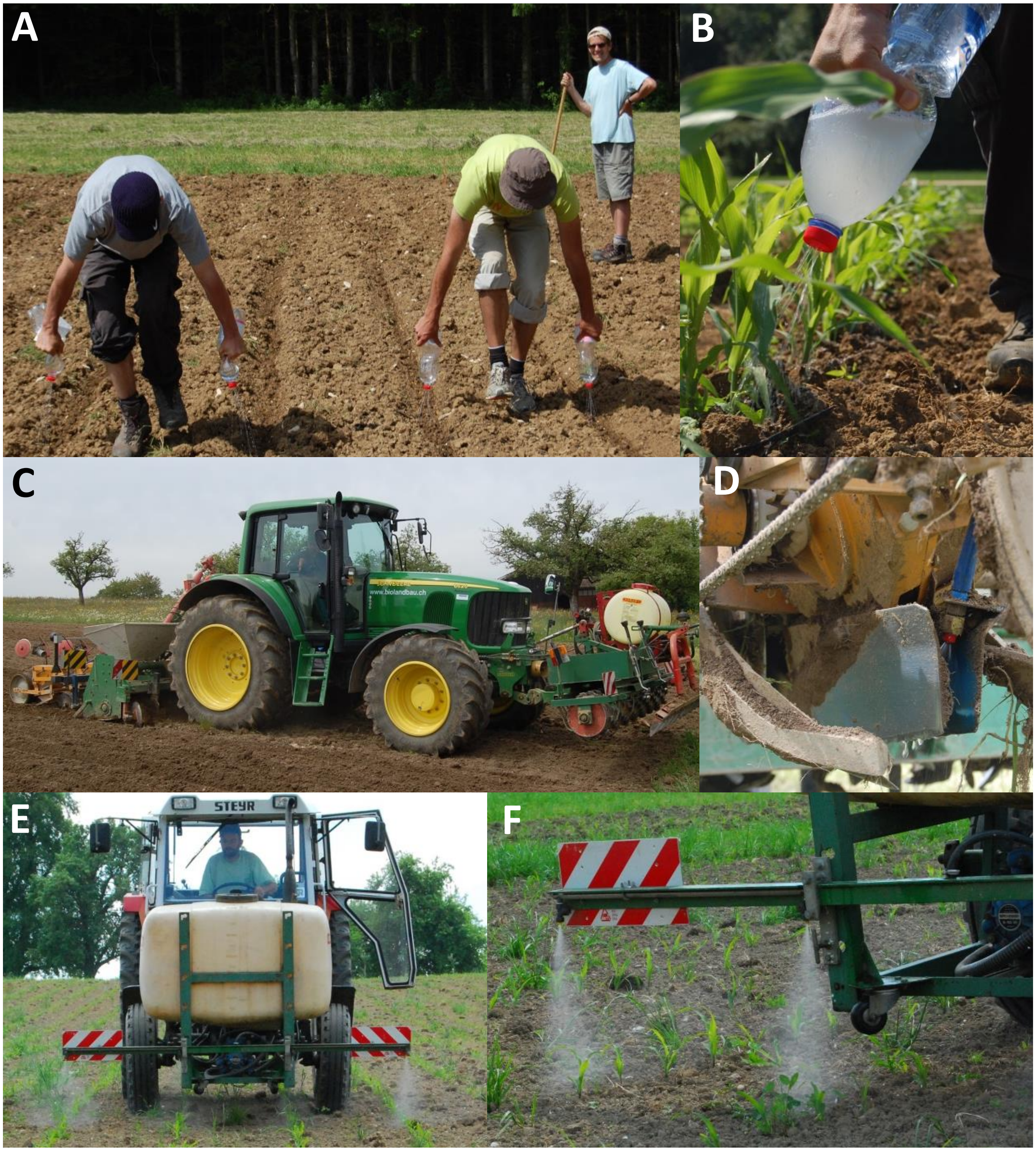

Figure S1 A) Manual application of bioeffectors (BEs) on top of the seeds before closing the seed furrows (1st application) and B) on top of the seed furrow (2nd application) at Buus site. At Hagenwil site, C) mechanical spray application of BEs on top of the seeds using a combined sowing machine D) with spray nozzles installed at the sowing blades (1st application) and E,F) on top of the row with a spray machine (2nd application).

2.2 Supplementary Tables

Table S1: Treatment effects on plant growth parameters assessed in the screening experiment conducted in the Buus soil under controlled conditions (experiment 1) according to Analysis of Variance (ANOVA). Degrees of freedom (DF), Sum of Squares (Sum Sq), Mean Squares (Mean Sq), p-value (Pr(>F)).

Response	Source of variation	DF	Sum Sq	Mean Sq	F value	Pr(>F)
Plant height (cm)	Bioeffector (BE)	2	260.137	130.069	3.979	0.021
	Additive	1	1.351	1.351	0.041	0.839
	Fertilization	5	15142.453	3028.491	92.637	0.000
	BE:Additive	2	117.947	58.973	1.804	0.169
	BE:Fertilization	10	243.407	24.341	0.745	0.681
	Additive:Fertilization	5	87.063	17.413	0.533	0.751
	BE:Additive:Fertilization	10	495.931	49.593	1.517	0.143
	Residuals	111	3628.823	32.692	---	---
Stem diameter (cm)	BE	2	2.653	1.327	3.381	0.038
	Additive	1	0.833	0.833	2.123	0.148
	Fertilization	5	164.863	32.973	84.025	0.000
	BE:Additive	2	1.268	0.634	1.616	0.203
	BE:Fertilization	10	2.042	0.204	0.520	0.873
	Additive:Fertilization	5	0.792	0.158	0.404	0.845
	BE:Additive:Fertilization	10	3.366	0.337	0.858	0.575
	Residuals	112	43.950	0.392	---	---
Shoot dry weight (g)	BE	2	0.757	0.378	6.404	0.002
	Additive	1	0.008	0.008	0.143	0.706
	Fertilization	5	19.283	3.857	65.262	0.000
	BE:Additive	2	0.229	0.114	1.936	0.149
	BE:Fertilization	10	0.590	0.059	0.998	0.450
	Additive:Fertilization	5	0.040	0.008	0.134	0.984
	BE:Additive:Fertilization	10	0.555	0.055	0.939	0.501
	Residuals	112	6.619	0.059	---	---
Root dry weight (g)	BE	2	0.575	0.288	18.761	0.000
	Additive	1	0.001	0.001	0.086	0.769
	Fertilization	5	2.427	0.485	31.670	0.000
	BE:Additive	2	0.071	0.035	2.312	0.104
	BE:Fertilization	10	0.136	0.014	0.887	0.547
	Additive:Fertilization	5	0.022	0.004	0.292	0.917
	BE:Additive:Fertilization	10	0.202	0.020	1.318	0.229
	Residuals	112	1.717	0.015	---	---

Table S2: Mean comparison of plant growth parameters with Bonferroni-adjusted p-values for the screening experiments conducted in the Buus soil under controlled conditions (experiment 1). Standard error (SE), Degrees of freedom (DF).

Contrast	Eestimate	SE	DF	t-ratio	p-value	Response
Proradix – No bioeffector (noBE)	1.908	1.163	111	1.640	0.311	Plant height (cm)
BEmix - noBE	0.523	1.163	111	0.449	1.000	
BEmix - Proradix	-1.385	1.167	111	-1.187	0.713	
Proradix - noBE	0.206	0.127	112	1.630	0.318	Stem diameter (cm)
BEmix - noBE	-0.002	0.127	112	-0.016	1.000	
BEmix - Proradix	-0.208	0.128	112	-1.629	0.318	
Proradix - noBE	0.138	0.049	112	2.800	0.018	Shoot dry weight (g)
BEmix - noBE	0.047	0.049	112	0.959	1.000	
BEmix - Proradix	-0.090	0.050	112	-1.822	0.213	
Proradix - noBE	0.062	0.025	112	2.483	0.044	Root dry weight (g)
BEmix - noBE	-0.093	0.025	112	-3.707	0.001	
BEmix - Proradix	-0.155	0.025	112	-6.125	<0.001	

Table S3: Treatment effects on plant growth parameters assessed in the screening experiment conducted in the Dompierre soil under controlled conditions (experiment 2) according to Analysis of Variance (ANOVA). Degrees of freedom (DF), Sum of Squares (Sum Sq), Mean Squares (Mean Sq), p-value (Pr(>F)).

Response	Source of variation	DF	Sum Sq	Mean Sq	F value	Pr(>F)
Plant height (cm)	Bioeffector (BE)	2	260.137	130.069	3.979	0.021
	Additive	1	1.351	1.351	0.041	0.839
	Fertilization	5	15142.453	3028.491	92.637	0.000
	BE:Additive	2	117.947	58.973	1.804	0.169
	BE:Fertilization	10	243.407	24.341	0.745	0.681
	Additive:Fertilization	5	87.063	17.413	0.533	0.751
	BE:Additive:Fertilization	10	495.931	49.593	1.517	0.143
	Residuals	111	3628.823	32.692	---	---
Stem diameter (cm)	BE	2	2.653	1.327	3.381	0.038
	Additive	1	0.833	0.833	2.123	0.148
	Fertilization	5	164.863	32.973	84.025	0.000
	BE:Additive	2	1.268	0.634	1.616	0.203
	BE:Fertilization	10	2.042	0.204	0.520	0.873
	Additive:Fertilization	5	0.792	0.158	0.404	0.845
	BE:Additive:Fertilization	10	3.366	0.337	0.858	0.575
	Residuals	112	43.950	0.392	---	---
Shoot dry weight (g)	BE	2	0.757	0.378	6.404	0.002
	Additive	1	0.008	0.008	0.143	0.706
	Fertilization	5	19.283	3.857	65.262	0.000
	BE:Additive	2	0.229	0.114	1.936	0.149
	BE:Fertilization	10	0.590	0.059	0.998	0.450
	Additive:Fertilization	5	0.040	0.008	0.134	0.984
	BE:Additive:Fertilization	10	0.555	0.055	0.939	0.501
	Residuals	112	6.619	0.059	---	---
Root dry weight (g)	BE	2	0.575	0.288	18.761	0.000
	Additive	1	0.001	0.001	0.086	0.769
	Fertilization	5	2.427	0.485	31.670	0.000
	BE:Additive	2	0.071	0.035	2.312	0.104
	BE:Fertilization	10	0.136	0.014	0.887	0.547
	Additive:Fertilization	5	0.022	0.004	0.292	0.917
	BE:Additive:Fertilization	10	0.202	0.020	1.318	0.229
	Residuals	112	1.717	0.015	---	---

Table S4: Mean comparison of maize shoot dry weight with Bonferroni-adjusted p-values for the screening experiment conducted in the Dompierre soil under controlled conditions (experiment 2). Standard error (SE), Degrees of freedom (DF).

Contrast	Additive	Fertilization	estimate	SE	DF	t-ratio	p-value
Proradix - no bioeffector (noBE)	No additive (A0)	No phosphorus (NoP)	0.291	0.121	112	2.399	0.054
BEmix - noBE	A0	NoP	-0.001	0.121	112	-0.009	1.000
BEmix - Proradix	A0	NoP	-0.293	0.140	112	-2.085	0.118
Proradix - noBE	Humic acids (HA)	NoP	0.753	0.140	112	5.364	<0.001
BEmix - noBE	HA	NoP	-0.025	0.140	112	-0.178	1.000
BEmix - Proradix	HA	NoP	-0.778	0.140	112	-5.542	<0.001
Proradix - noBE	A0	Rock phosphate (RP)	0.682	0.140	112	4.865	<0.001
BEmix - noBE	A0	RP	0.037	0.140	112	0.267	1.000
BEmix - Proradix	A0	RP	-0.645	0.140	112	-4.598	<0.001
Proradix - noBE	HA	RP	-0.395	0.140	112	-2.816	0.017
BEmix - noBE	HA	RP	-0.303	0.140	112	-2.156	0.100
BEmix - Proradix	HA	RP	0.092	0.140	112	0.659	1.000
Proradix - noBE	A0	Compost	0.174	0.140	112	1.243	0.649
BEmix - noBE	A0	Compost	0.109	0.140	112	0.780	1.000
BEmix - Proradix	A0	Compost	-0.065	0.140	112	-0.463	1.000
Proradix - noBE	HA	Compost	-0.875	0.140	112	-6.237	<0.001
BEmix - noBE	HA	Compost	-0.655	0.140	112	-4.669	<0.001
BEmix - Proradix	HA	Compost	0.220	0.140	112	1.568	0.359
Proradix - noBE	A0	Digestate	-0.262	0.140	112	-1.866	0.194
BEmix - noBE	A0	Digestate	0.151	0.140	112	1.074	0.855
BEmix - Proradix	A0	Digestate	0.412	0.140	112	2.941	0.012
Proradix - noBE	HA	Digestate	-0.152	0.140	112	-1.087	0.838
BEmix - noBE	HA	Digestate	-0.175	0.140	112	-1.247	0.644
BEmix - Proradix	HA	Digestate	-0.023	0.140	112	-0.160	1.000
Proradix - noBE	A0	Farmyard manure (FYM)	-0.282	0.140	112	-2.014	0.139
BEmix - noBE	A0	FYM	-0.005	0.140	112	-0.036	1.000
BEmix - Proradix	A0	FYM	0.277	0.140	112	1.978	0.151
Proradix - noBE	HA	FYM	0.732	0.140	112	5.222	<0.001
BEmix - noBE	HA	FYM	0.602	0.140	112	4.295	<0.001
BEmix - Proradix	HA	FYM	-0.130	0.140	112	-0.927	1.000
Proradix - noBE	A0	Pellets	0.243	0.140	112	1.729	0.260
BEmix - noBE	A0	Pellets	0.250	0.140	112	1.782	0.232
BEmix - Proradix	A0	Pellets	0.007	0.140	112	0.053	1.000
Proradix - noBE	HA	Pellets	0.768	0.140	112	5.471	<0.001
BEmix - noBE	HA	Pellets	0.797	0.140	112	5.685	<0.001
BEmix - Proradix	HA	Pellets	0.030	0.140	112	0.214	1.000

Table S5: Treatment effects on plant growth parameters assessed in the eight-week experiment conducted in the Buus soil under controlled conditions (experiment 3) according to Analysis of Variance (ANOVA). Degrees of freedom (DF), Sum of Squares (Sum Sq), Mean Squares (Mean Sq), p-value (Pr(>F)).

Response	Source of variation	DF	Sum Sq	Mean Sq	F value	Pr(>F)
Plant height (cm)	Bioeffector (BE)	1	8.100	8.100	0.214	0.646
	Additive	1	99.225	99.225	2.627	0.115
	Fertilization	1	864.900	864.900	22.898	<0.001
	BE:Additive	1	24.025	24.025	0.636	0.431
	BE:Fertilization	1	0.900	0.900	0.024	0.878
	Additive:Fertilization	1	2.025	2.025	0.054	0.818
	BE:Additive:Fertilization	1	60.025	60.025	1.589	0.217
	Residuals	32	1208.700	37.772	---	---
Shoot phosphorus (P) uptake (mg P)	BE	1	3.850	3.850	1.120	0.298
	Additive	1	3.358	3.358	0.977	0.330
	Fertilization	1	4898.033	4898.033	1424.988	<0.001
	BE:Additive	1	1.395	1.395	0.406	0.529
	BE:Fertilization	1	9.497	9.497	2.763	0.106
	Additive:Fertilization	1	0.107	0.107	0.031	0.861
	BE:Additive:Fertilization	1	1.139	1.139	0.331	0.569
	Residuals	32	109.992	3.437	---	---
Shoot dry weight (g)	BE	1	0.224	0.224	0.076	0.785
	Additive	1	2.426	2.426	0.823	0.371
	Fertilization	1	928.429	928.429	315.022	<0.001
	BE:Additive	1	3.875	3.875	1.315	0.260
	BE:Fertilization	1	14.677	14.677	4.980	0.033
	Additive:Fertilization	1	0.184	0.184	0.062	0.804
	BE:Additive:Fertilization	1	0.002	0.002	0.001	0.979
	Residuals	32	94.310	2.947	---	---
Root dry weight (g)	BE	1	0.443	0.443	0.867	0.359
	Additive	1	0.322	0.322	0.631	0.433
	Fertilization	1	85.293	85.293	166.985	<0.001
	BE:Additive	1	2.021	2.021	3.956	0.055
	BE:Fertilization	1	1.403	1.403	2.746	0.107
	Additive:Fertilization	1	0.776	0.776	1.518	0.227
	BE:Additive:Fertilization	1	0.000	0.000	0.000	0.988
	Residuals	32	16.345	0.511	---	---

Table S6: Mean comparison with Bonferroni-adjusted p-values for *Pseudomonas* strain DSMZ 13134 abundance (colony forming units / mg root dry weight) in the time series experiment conducted in the Buus soil under controlled conditions (experiment 3b). Standard error (SE), Degrees of freedom (DF).

Contrast	Ratio	SE	DF	null	t-ratio	p-value
week2 / week1	0.006	0.01	10	1	-3.999	0.01
week3 / week2	0.243	0.31	10	1	-1.095	1.00
week4 / week3	3.406	4.40	10	1	0.948	1.00
week5 / week4	0.084	0.11	10	1	-1.911	0.34

Table S7: Treatment effects on plant growth parameters assessed in the eight-week experiment conducted in the Dompierre soil under controlled conditions (experiment 4) according to Analysis of Variance (ANOVA). Degrees of freedom (DF), Sum of Squares (Sum Sq), Mean Squares (Mean Sq), p-value (Pr(>F)).

Response	Source of variation	DF	Sum Sq	Mean Sq	F value	Pr(>F)
Plant height (cm)	Additive	1	0.156	0.156	0.002	0.961
	Fertilization	3	38148.369	12716.123	198.486	<0.001
	Additive:Fertilization	3	17.069	5.690	0.089	0.966
	Residuals	32	2050.100	64.066	---	---
Shoot phosphorus (P) uptake (mg P)	Additive	1	0.002	0.002	0.388	0.538
	Fertilization	3	31.557	10.519	2531.881	<0.001
	Additive:Fertilization	3	0.015	0.005	1.189	0.330
	Residuals	32	0.133	0.004	---	---
Shoot dry weight (g)	Additive	1	4.238	4.238	2.178	0.150
	Fertilization	3	7350.568	2450.189	1259.171	<0.001
	Additive:Fertilization	3	5.525	1.842	0.946	0.430
	Residuals	32	62.268	1.946	---	---
Root dry weight (g)	Additive	1	0.018	0.018	1.551	0.222
	Fertilization	3	24.753	8.251	702.107	<0.001
	Additive:Fertilization	3	0.024	0.008	0.686	0.567
	Residuals	32	0.376	0.012	---	---

Table S8: Treatment effects on soil parameters assessed in the soil incubation experiment conducted in the Dompierre soil under controlled conditions according to Analysis of Variance (ANOVA). Degrees of freedom (DF), Sum of Squares (Sum Sq), Mean Squares (Mean Sq), p-value (Pr(>F)).

Response	Source of variation	DF	Sum Sq	Mean Sq	F value	Pr(>F)
Soil pH	Fertilization	3	0.085	0.028	4.936	0.008
	Additive	1	0.002	0.002	0.371	0.548
	Fertilization:Additive	3	0.028	0.009	1.625	0.210
	Residuals	24	0.138	0.006	---	---
Basal respiration (ugCO2-C /kg soil)	Fertilization	3	137.113	2.042	1299.834	<0.001
	Additive	1	0.376	0.002	1.507	0.232
	Fertilization:Additive	3	1.002	0.007	4.523	0.012
	Residuals	24	2.379	0.002	---	---
Resin phosphorous (P) (mg resinP/kg soil)	Fertilization	3	137.113	45.704	461.047	<0.001
	Additive	1	0.376	0.376	3.798	0.063
	Fertilization:Additive	3	1.002	0.334	3.371	0.035
	Residuals	24	2.379	0.099	---	---

Table S9: Linear mixed effect model for plant (maize) related data as assessed in the field experiment (Buus 1). Numerator degree of freedom (numDF), denomenator -degree of freedom (denDF).

Response	Source of variation	numDF	denDF	F-value	p-value
Plant height	Intercept	1	35	38.747	<0.001
	Bioeffector (BE)	2	35	0.145	0.865
	Fertilization	1	35	0.858	0.361
	BE:Fertilization	2	35	0.062	0.940
Shoot dry weight (DW) (t/ha)	Intercept	1	35	38.975	<0.001
	BE	2	35	0.069	0.933
	Fertilization	1	35	0.013	0.910
	BE:Fertilization	2	35	0.210	0.812
Phosphorous (P) uptake (kg P/ha)	Intercept	1	35	97.382	<0.001
	BE	2	35	0.367	0.695
	Fertilization	1	35	0.021	0.885
	BE:Fertilization	2	35	0.029	0.972
P concentration (mgP/g DW)	Intercept	1	35	281.257	<0.001
	BE	2	35	0.424	0.658
	Fertilization	1	35	0.550	0.463
	BE:Fertilization	2	35	0.621	0.543
Number of plants (plant/m2)	Intercept	1	35	638.789	<0.001
	BE	2	35	0.355	0.704
	Fertilization	1	35	0.057	0.812
	BE:Fertilization	2	35	0.424	0.658
Number of ears (ears/m2)	Intercept	1	35	267.753	<0.001
	BE	2	35	0.269	0.766
	Fertilization	1	35	0.115	0.736
	BE:Fertilization	2	35	0.041	0.960

Table S10: Linear mixed effect model for plant (maize) related data as assessed in the field experiment (Buus 2). Numerator degree of freedom (numDF), denomenator -degree of freedom (denDF).

Response	Source of variation	numDF	denDF	F-value	p-value
Plant height (cm)	Intercept	1	15	4536.380	<0.001
	Bioeffector (BE)	2	15	0.756	0.487
	Fertilization	1	15	0.229	0.639
	BE:Fertilization	2	15	4.821	0.024
Shoot dry weight (DW) (t/ha)	Intercept	1	15	809.784	<0.001
	BE	2	15	0.546	0.590
	Fertilization	1	15	0.635	0.438
	BE:Fertilization	2	15	3.649	0.051
Phosphorous (P) uptake (kg P/ha)	Intercept	1	15	276.659	<0.001
	BE	2	15	0.256	0.777
	Fertilization	1	15	0.075	0.787
	BE:Fertilization	2	15	1.251	0.314
P concentration (mgP/g DW)	Intercept	1	15	1397.807	<0.001
	BE	2	15	0.097	0.909
	Fertilization	1	15	0.333	0.573
	BE:Fertilization	2	15	0.339	0.718
Number of plants (plant/m2)	Intercept	1	15	3140.260	<0.001
	BE	2	15	0.779	0.476
	Fertilization	1	15	0.155	0.700
	BE:Fertilization	2	15	1.356	0.287
Number of ears (ears/m2)	Intercept	1	15	1550.794	<0.001
	BE	2	15	1.340	0.291
	Fertilization	1	15	0.076	0.786
	BE:Fertilization	2	15	1.968	0.174

Table S11: Mean comparison with Bonferroni-adjusted p-values of maize height in the field experiment Buus 2. Standard error (SE), Degrees of freedom (DF).

Contrast	Fertilisation	Estimate	SE	DF	t-ratio	p-value
No bioeffector (NoBE) - Nematec	No phosphorus (No P)	0.033	0.069	15	0.468	1.000
Proradix - Nematec		0.110	0.069	15	1.586	0.401
Proradix - NoBE		0.078	0.069	15	1.117	0.845
NoBE - Nematec	Pellets	-0.150	0.069	15	-2.162	0.142
Proradix - Nematec		-0.193	0.069	15	-2.775	0.043
Proradix - NoBE		-0.043	0.069	15	-0.613	1.000

Table S12: Linear mixed effect model for plant (maize) related data as assessed in the field experiment at the Hagenwil site. Numerator degree of freedom (numDF), denomenator -degree of freedom (denDF).

Response	Source of variation	numDF	denDF	F-value	p-value
Corn yield (t/ha)	Intercept	1	46	25.372	<0.001
	Bioeffector (BE)	2	46	0.546	0.583
Corn yield per plant (g/plant)	Intercept	1	46	125.774	<0.001
	BE	2	46	2.141	0.129
Number of plants (plants/m^2)	Intercept	1	46	30.078	<0.001
	BE	2	46	1.119	0.335
Number of ears (ears/m^2)	Intercept	1	46	31.830	<0.001
	BE	2	46	1.843	0.170
